# Genotypic and Phenotypic Diversity Does Not Affect Productivity and Drought Response in Competitive Stands of *Trifolium repens*

**DOI:** 10.3389/fpls.2016.00364

**Published:** 2016-03-29

**Authors:** Heidrun Huber, Heinjo J. During, Fabienne Bruine de Bruin, Peter J. Vermeulen, Niels P. R. Anten

**Affiliations:** ^1^Department of Experimental Plant Ecology, Institute for Water and Wetland Research, Radboud University NijmegenNijmegen, Netherlands; ^2^Section of Ecology and Biodiversity, Institute of Environmental Biology, Utrecht UniversityUtrecht, Netherlands; ^3^Centre for Crop Systems Analysis, Wageningen UniversityWageningen, Netherlands

**Keywords:** clonal growth, competition, drought, genotypic selection, genetic and phenotypic diversity, size hierarchies, *Trifolium repens*

## Abstract

Clonal plants can form dense canopies in which plants of different genetic origin are competing for the uptake of essential resources. The competitive relationships among these clones are likely to be affected by extreme environmental conditions, such as prolonged drought spells, which are predicted to occur more frequently due to global climate change. This, in turn, may alter characteristics of the ecological system and its associated functioning. We hypothesized that the relative success of individual clones will depend on the size of the ramets as ramets with larger leaves and longer petioles (large ramets) were predicted to have a competitive advantage in terms of increased light interception over smaller-sized ramets. Under drier conditions the relative performances of genotypes were expected to change leading to a change in genotype ranking. We also hypothesized that increased genotypic and phenotypic diversity will increase stand performance and resistance to drought. These hypotheses and the mechanisms responsible for shifts in competitive relationships were investigated by subjecting genotypes of the important pasture legume *Trifolium repens* to competition with either genetically identical clones, genetically different but similarly sized clones, or genetically as well as morphologically different clones under well-watered and dry conditions. Competitive relationships were affected by ramet size with large genotypes outperforming small genotypes in diverse stands in terms of biomass production. However, large genotypes also produced relatively fewer ramets than small genotypes and could not benefit in terms of clonal reproduction from competing with smaller genotypes, indicating that evolutionary shifts in genotype composition will depend on whether ramet size or ramet number is under selection. In contrast to our hypotheses, diversity did not increase stand performance under different selection regimes and genotype ranking was hardly affected by soil moisture, indicating that increasing fluctuations in water availability result in few short-term effects on genotypic diversity in this stoloniferous grassland species. Communities dominated by stoloniferous herbs such as *T. repens* may be relatively resilient to environmental change and to low levels of genetic diversity.

## Introduction

Ecosystem productivity and resilience to environmental fluctuations are generally believed to increase with increasing diversity (Cardinale et al., 2013; de Mazancourt et al., 2013; Gross et al., 2014). As different species can occupy different ecological niches, complex interactions of different trophic levels exist and negative feedback loops driven by, e.g., soil pathogens are less likely to affect species performance in diverse ecosystems as large species- or genotype-specific pathogen populations rarely build up (Hendriks et al., 2013; van der Putten et al., 2013; Bardgett and van der Putten, 2014; Brotherton and Joyce, 2015). While these concepts have long been applied to species diversity, increasing evidence exists that within-species genetic diversity can be equally important (Reusch et al., 2005; Hughes et al., 2008; Ellers et al., 2011; Grettenberger and Tooker, 2015). The effects of intraspecific genetic diversity may be the consequence of different processes that enable genotypes to occupy slightly different niches, e.g., by association with different microbial communities or herbivores (Ofek-Lalzar et al., 2014; Simonsen et al., 2014; Grettenberger and Tooker, 2015) or due to variation in other functional traits (Ellers et al., 2011; Hughes, 2014). Increased persistence under and resilience to environmental stress in genotypically diverse populations would require among-genotype variation in performance under different selection regimes, as different genetically determined traits interact in their specific way with the environment depending on the specific trait values, ultimately leading to a change in the relative abundance of genotypes (Hughes et al., 2008; Engelhardt et al., 2014). However, the interactions between genotypic and functional diversity and environmental fluctuations are largely unresolved to date.

Species capable of vegetative propagation are an important component in most herbaceous plant communities (Schmid and Harper, 1985; Hamilton et al., 1987; van Groenendael et al., 1996). In contrast to species relying solely on sexual reproduction, vegetatively reproducing species can maintain, or even multiply specific genotypes over prolonged periods of time. Seed set is usually associated with genetic recombination, leading to a new set of genotypes characterized by new trait combinations. While sexual reproduction is one of the preconditions creating the necessary variation for selection to act upon, it may also slow down or weaken the response to selection and adaptation to changed environmental conditions as the genes are reshuﬄed each generation (Becks and Agrawal, 2012). Clonal species, however, do not require flowering and seed production for population maintenance. Clonal propagation can therefore be expected to lead to fast evolutionary shifts if, in a genetically diverse population, variation in relative fitness among different genotypes exists (Gladieux et al., 2015).

Changes in environmental conditions are bound to alter the consequences of trait variation on competitive outcomes, thereby leading to shifts in genotype performance (Stuefer et al., 2009). In clonal plant populations short-term responses of genetic diversity may thus be enhanced as clonal propagation allows for relatively fast and specific selection for the best adapted genotypes. However, this may also lead to reduced genetic and phenotypic diversity within populations if selection pressures are strong, environmental changes slow and input from new genetic material by sexual reproduction low. This in turn could potentially reduce the ability to adapt to new environmental challenges. Alternatively, environmental effects on genotypic diversity may be mitigated through high levels of phenotypic plasticity, enabling multiple genotypes to buffer environmental fluctuations and maintain genetically diverse populations over time (Nicotra et al., 2010); or because genotypes have constitutively different relative performance in different environmental conditions. As changes in genetic diversity within populations can translate into variation at higher trophic levels (Reusch et al., 2005; Barton et al., 2015), whether environmental fluctuations maintain diversity or not can have a large influence on community processes.

Over the last decades environmental conditions are increasingly changing, in many parts of the world leading to a higher frequency of extreme weather conditions such as early or prolonged drought events (IPCC, 2014). Drought conditions may, directly or indirectly, select for a different suite of traits as compared to well-watered conditions. Profuse growth under favorable soil conditions will lead to asymmetric competition for light (Anten and Hirose, 2001). In the common stoloniferous species *Potentilla reptans* taller, heavier leaves placed at the top of the canopy captured a disproportionate amount of light per unit invested biomass compared to leaves with a lower investment that were placed lower down (Vermeulen et al., 2008b). Hence in competition, genotypes with large ramets can maintain their position in high light conditions through their high investment in leaves, while genotypes that produce smaller ramets can have a strongly reduced productivity due to shading if the height growth of the competitive surrounding is fast enough (Vermeulen et al., 2008a). Because there is a trade-off between investment in large ramets and the number of ramets (Huber and Wiggerman, 1997; Vermeulen and During, 2010), competition for light should lead to selection for genotypes producing large ramets with long petioles, but with a lower number of ramets.

The strong selective pressure on aboveground competitive ability will become lower when water shortage leads to reduced biomass development. Under such conditions, selection can be expected to be more strongly driven by belowground competition, which is usually assumed to be less dependent on size, i.e., symmetric (Schwinning and Weiner, 1998; Casper et al., 2003). In addition, selection gradients on leaf size were found to differ between low and high water availability in *Cakile edentula* (Dudley, 1996). This leads to the prediction that depending on the soil water availability different genotypes will be selected for, depending on their specific suite of traits and the potential to show adaptive responses to the respective environmental conditions.

In this study, we artificially manipulated genetic and functional diversity, by creating genetically homogeneous stands, genetically diverse, but phenotypically similar stands and phenotypically as well as genetically diverse stands. By using genotypes that differed up to 2.5-fold in the ecologically relevant key-trait ramet size we manipulated the potential range of phenotypic diversity within the experimental plots. The study was performed to test (I) the effects of water availability and phenotypic characteristics on relative genotype performance, (II) the consequence of inherent genetic variation in trait expression for phenotypic variation in competitive stands, and (III) the consequence of genetic and phenotypic variation for population performance.

(I) We expected shifts in genotype abundance, and the direction of the shift to be dependent on water availability. The relative shift in genotype abundance was hypothesized to be driven by the phenotypic characteristics of the genotypes. Generally we expected genotypes characterized by large ramets to be competitively superior over genotypes characterized by smaller ramets, due to different positioning of leaves within the canopy. This relative advantage was expected to be reduced under drought conditions, as overall leaf area of the stands will be smaller and light gradients less steep. (II) We expected that competition would lead to shifts in variation in trait expression. Variation could decrease due to more uniform trait expression, if e.g., all petioles assume a similar length in competitive stands, or, alternatively, could increase due to asymmetric competition having stronger negative effects on genotypes which are positioned lower within a size hierarchy. (III) We also hypothesized that genetically and phenotypically diverse stands would have a higher overall performance and be more resistant (Grimm and Wissel, 1997) to drought as different genotypes may differ in their drought tolerance.

## Materials and Methods

### Plant Material

The experiment was performed with the clonal species *Trifolium repens*. This species grows by means of aboveground monopodial stolons along which single-leaved rooted ramets are positioned at regular distances. The plants can produce extended integrated clonal systems which can face high intensity of competition at different organizational levels. Each ramet consists of an internode, a leaf, one axillary meristem which can give rise to either a flower or a new lateral stolon, and roots (Hutchings et al., 1997; Huber and During, 2000). The size of the modular units (ramets) can vary greatly among genotypes, which is of pivotal importance for the relative performance of the genotypes in their respective environments (Weijschedé et al., 2006, 2008). Ramet size reflects an internally determined covariation of leaf size, internode length, and petiole length, with a positive correlation among these traits (Weijschedé et al., 2006). Previous research has shown that *T. repens* is characterized by a high genetically determined variability of trait expression, strongly responds to competition and that this response may be different depending on whether the competitors are from the same genotype or genetically and morphologically different (Turkington and Burdon, 1983; Hutchings et al., 1997; Lotscher and Hay, 1997; Bittebiere et al., 2012). In addition, in this species leaf size has been shown to differently affect drought tolerance and recovery to drought (Annicchiarico and Piano, 2004; Li et al., 2013). The formation of dense communities under natural conditions with high incidence of inter- and intra-clonal competition, high levels of plasticity, fast ramet production rates and large inter-genotypic variation makes *T. repens* an ideal species to experimentally study the effect of genetic and functional diversity on population performance.

For the present experiments eight genotypes differing in ramet size were selected from 36 genotypes used by Weijschedé et al. (2006, 2008). The genotypes used in this experiment were originally collected in a riverine grassland along the river Waal (Ewijk, the Netherlands). Distance between plants was at least 5 m and plants were identified as being genetically different by means of AFLP analyses (J.L. Peters, unpublished results; Supplementary Figure 1 in Supplementary Data Sheet 1). We chose four genotypes characterized by small ramets and four genotypes characterized by large ramets (**Table 1**). The choice of the genotypes was primarily based on petiole and leaf length, as these traits are important for determining the competitive hierarchies in dense stands (Weiner, 1985; Vermeulen et al., 2008a; Weijschedé et al., 2008).

**Table 1:** Phenotypic ramet characteristics of genotypes used in this experiment.

Genotype identifier	Size class	Petiole length (mm)	Leaf length (mm)	Internode length (mm)
A25 ◯	Large	53.8	11.8	14.5
A4 ●	Large	48.5	13.2	21.9
B15 ▼	Large	52.7	16.5	22.9
B4 △	Large	49.5	14.3	19.3
B7 ■	Small	18.8	8.7	13.7
D18 □	Small	22.1	8.0	15.8
D21 ◆	Small	20.8	8.5	13.5
D30 ◇	Small	22.2	9.4	12.3
**Average initial variation**				
Phenotypically similar		6.2	10.5	14.8
Phenotypically diverse		48.5	29.3	24.9

### Experiment

**FIGURE 1:**
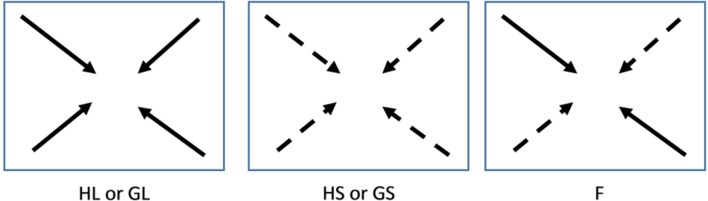
**Schematic drawing of experimental set up.** Four lateral cuttings consisting of approximately five ramets were planted 5 cm from the corners of the trays and the stolons of the plants were directed toward the center of the trays. H: genetically and phenotypically homogeneous monostands; G: genetically diverse but phenotypically similar stands; F: phenotypically diverse stands; S and dashed arrows: Small size class; L and solid arrows: Large size class. A total of 768 plants were used for the experiment. Number of replicates per genotype per treatment: HL and HS: 24; GL, GS, and F: 12

Genotypes were collected more than 5 years prior to the experiment to minimize potential environmentally induced carry-over effects. During summer genotypes were maintained under outdoor conditions in the experimental garden of Nijmegen University in containers (l × w × h = 0.4 m × 0.3 m × 0.3 m) filled with a 1:1 mixture of commercial potting compost and sand with an addition of 4gr slow release fertilizer (Osmocote+, Scott Sierra International B.V. Heerlen, the Netherlands, 9–10 months) per liter soil. Plants were watered regularly and repotted each year into new containers. During winter plants were moved to a heated greenhouse where the experiment was also performed. Incident light was supplemented by high pressure sodium lamps (Hortilux Schreder, 600 Watt) whenever light availability fell below 400 μmol m^-2^ s^-1^ between 6:00 h and 22:00 h. Before the experiment, 16 lateral cuttings (hereafter referred to as clonal fragments) containing one rooted ramet with a lateral stolon consisting of three to five newly produced ramets were made for each genotype. The cuttings were distributed over 32 trays (l × w × h = 0.22 m × 0.18 m × 0.05 m) filled with 1 l substrate consisting of five parts loamy sand and one part sieved potting compost. Three grams slow release fertilizer (Osmocote+ Scott Sierra International, 3–4 months) were added to each tray. Trays were filled and kept wet for 2 weeks before the onset of the experiment for nutrient release to commence prior to the experiment. Each tray contained four clonal fragments. Each clonal fragment was planted in one of the corners of the trays, with the rooted ramets approximately 5 cm from the corner and the stolon apices directed toward the center of the tray to facilitate competition among the four plants (**Figure 1**). Throughout the experiment the stolons were prevented from growing out of the trays by bending them back to the tray in order to increase competitive interactions within the trays and avoid support of clones in the trays by ramets subjected to favorable light conditions outside the tray borders.

The clonal fragments were subjected to three types of competition treatments, in which they were allowed to compete above- as well as belowground (**Figure 1**). In the monostands four clonal fragments of the same genotype were competing with each other (one tray per genotype per block). In the phenotypically similar stands clonal fragments of either the four small or the four large genotypes were competing with each other. This treatment was repeated twice per size class per block. In the phenotypically diverse stands two small and two large genotypes were grown in each tray. This treatment was repeated four times per block with a different combination of two large and two small genotypes in each tray. Each genotype was thus used twice with three different competitors. In this treatment, the two large or the two small genotypes were grown in opposite corners, resulting in each phenotype having two neighbors of the opposite phenotype. All combinations were subjected to either well-watered (moist) or drought (dry) conditions. For the well-watered conditions soil moisture was kept at 35% (v:v) and for the drought treatments soil moisture was kept at 10–15% (v:v). The volumetric water content in the soil was monitored every other day by measuring at four different locations in each tray using a theta probe (HH2, Moisture Meter version 2; Delta-T Devices, Cambridge, UK) after which the trays were supplemented with water to regain 35% and 15% soil moisture, respectively. Drought treatments started 2 weeks after planting and lasted for 4 weeks. The experiment took place in the greenhouse of Nijmegen University in the period April–June. Treatments were repeated in six temporal blocks starting at 1-week intervals. The experiment contained a total number of 192 trays and 768 plants. For the monocultures a total of 24 clonal fragments per genotype were used (spread over six trays) and for the phenotypically similar and phenotypically diverse stands a total of 12 clonal fragments were used per genotype (spread over 12 trays).

At harvest all plants were washed carefully free of substrate to allow for separation of the roots. As the stolon connections of *T. repens* are long-lived and were still intact at the end of the experiment, the four original plants could be separated. For each clonal fragment the length of the main stolon, number of ramets and lateral branches, total dry weight as well as allocation to roots, stolons and leaves were measured. In addition the fourth youngest ramet of the main stolon was harvested separately to determine ramet architecture (internode length, petiole length, and leaf size). To get information about leaf turnover, the number of leaves on the main stolon was counted as well. Plant parts were dried for at least 48 h at 72°C before determining dry weights.

### Statistical Analyses

Overall production of the trays in terms of total ramet number and biomass was analyzed by means of two-way ANOVA, with water availability and competition type as the main factors. Growth, ramet architecture and biomass allocation of individual clonal fragments were analyzed by means of a three-way nested ANOVA with competition treatment, genotype size class and soil moisture treatment as main effects. Genotypes were nested within size class and the temporal block was added as a random factor. As performing ANOVA’s on absolute trait values can impede the interpretation of biologically relevant relative trait responses to treatments in traits which are characterized by inherently different trait values (Huber, 1996) we also log-transformed ramet size to test whether small and large sized genotypes responded differently to moisture treatments.

In order to test whether plants assume similar phenotypes if grown in mixtures or, alternatively, phenotypic variation among plants gets reinforced due to, e.g., effects of size hierarchies, phenotypic diversity was determined by calculating the realized coefficient of variation (CV) of phenotypic traits among the four competing clonal fragments. A two-way ANOVA was performed on these CVs with competition treatment and moisture treatment as main effects to test whether phenotypic diversity was affected by the type of competition treatment and soil moisture conditions.

To test whether genetically and phenotypically diverse stands had a higher overall performance than can be expected on the basis of their monostands, we used the additive partitioning method (Loreau and Hector, 2001). This method calculates a net effect of treatments on biomass (ΔY) as the differences between observed yield and the expected yield of the mixture. The expected yield is defined as the average monostand yield of the genotypes in the mixture. Because of significant block effects, we used monostand within blocks to calculate this expected yield (see Van Ruijven and Berendse, 2009). ΔY was then decomposed into a complementarity effect (whether the genotype yields in mixtures are higher or lower than expected on the basis of the weighted average monostand yields) and a selection effect (the covariance between the monostand yield of the genotype and the difference between expected yield in monostand and the observed yield in the mixtures of the genotypes -ΔRY-). In addition, a multiple regression was performed for each moisture treatment separately. Total ramet number or biomass were the dependent variables and regressed against the CV of the different phenotypic characteristics.

We performed a correspondence analysis (CA) as implemented in PC-Ord 6.03 (option RA; McCune and Mefford, 2011) to get an overview of the results and insight into the mutual relationships between the measured traits in the face of the two water availability treatments and the three competition treatments. We analyzed the datasets of the measured traits of the plants in both water treatments separately and in combination, with the major directions of variation correlated to treatments, block effect, type of competition and individual genotypes calculated afterward and added as supplementary variables in the ordination graphs.

## Results

### Relative Genotype Abundance

**FIGURE 2:**
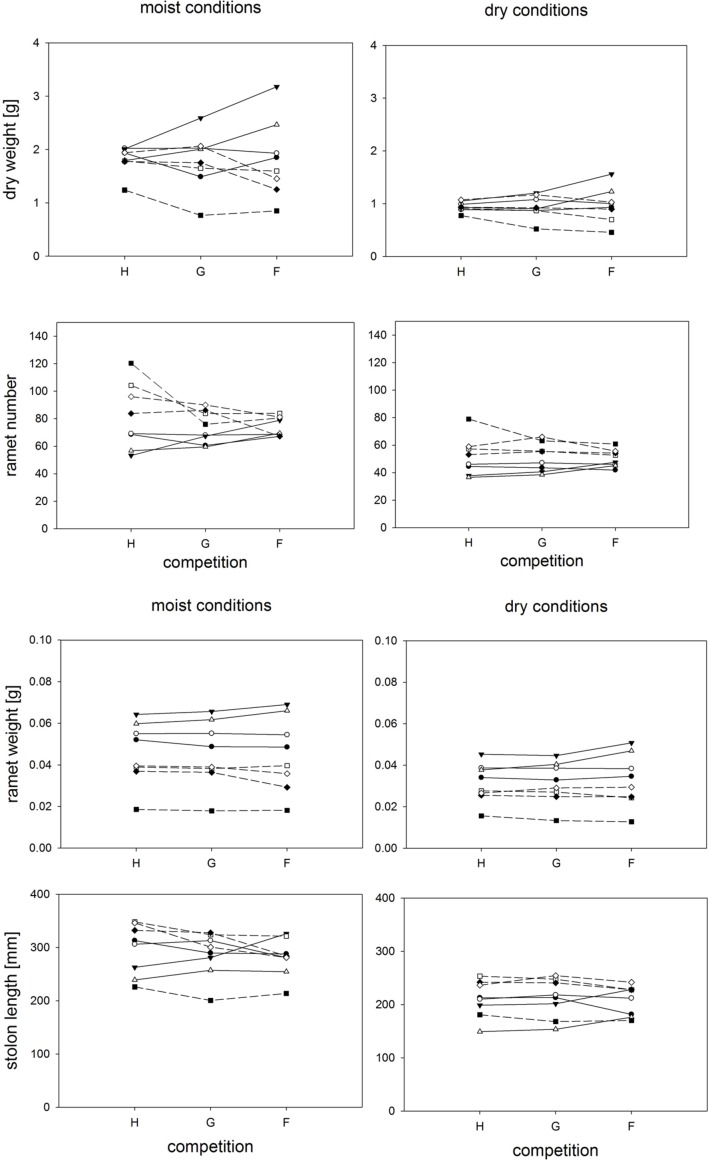
**Clonal fragments: Response of plant biomass, ramet number, average ramet weight, and stolon length to competition and soil moisture treatments.** Each line represents the mean value of a specific genotype in the respective treatments. Solid lines represent large genotypes, dashed lines small genotypes. Competition type abbreviations: H: homogeneous monostands; G: phenotypically similar stands; F: phenotypically diverse stands. For significances, see **Table 2**.

Neither competition type nor moisture treatments had strong effects on the genotype ranking, but the relative variation among genotypes with respect to total biomass or ramet number varied conspicuously among competition treatments (**Figure 2**, **Table 2**). Variation in plant weight was up to fourfold larger in the phenotypically diverse stands than in the other two competition treatments. The biomass of large and small plants responded differently to competition treatments (**Table 2**). This was mainly due to the positive response of the large sized genotypes B15 and B4 to increased diversity, and a negative response for most of the small genotypes in the phenotypically diverse treatment (**Figure 2**). Small-sized genotypes produced on average twice as many ramets as large-sized genotypes did. Contrary to the results on biomass, variation in ramet number was greater in monostands than in phenotypically diverse stands (**Figure 2**). The number of ramets produced by large and small genotypes responded differently to competition treatments (**Figure 2**, **Table 2**). The small-sized genotypes showed on average a stronger reduction in ramet number in response to diversity, while the large-sized genotypes showed a slight increase in ramet number in response to increasing diversity. In addition, within size classes there was significant variation in total ramet number in response to competition treatments, with the small-sized genotype B7 showing the strongest decrease and the large sized genotype B15 the strongest increase in ramet number in response to increasing diversity.

**Table 2:** Results (*F*-values and their significance) of a three-way nested ANOVA testing for the effects of competition treatment, genotype size class and soil moisture treatment on growth, ramet architecture and biomass allocation.

	df	Dry weight [g]	Ramet number [n]	Ramet weight[g]	Stolon length [mm]	Internode length [mm]	Petiole length [mm]	Leaf size [cm^2^]	Root allocation [%]	Stolon allocation [%]	Leaf allocation [%]
Competition (C)	2	0.3^ns^	0.2^ns^	0.2^ns^	0.9^ns^	0.7^ns^	0.4^ns^	1.1^ns^	0.2^ns^	0.1^ns^	0.1^ns^
Size class (S)	1	1.7^ns^	**41.7**^∗^	**17.4**^∗∗^	0.5^ns^	0.3^ns^	**23.8**^∗^	**37.4**^∗∗∗^	**28.6**^∗∗^	*4.4*^$^	0.1^ns^
C ^∗^ S	2	**5.1**^∗∗∗^	**4.7**^∗^	**4.1**^∗^	*3.1*^$^	*2.9*^$^	0.1^ns^	2.3^ns^	1.6^ns^	*3.4*^$^	1.4^ns^
Genotype(size)	6	**13.0**^∗∗∗^	1.5^ns^	**19.2**^∗∗∗^	**14.0**^∗∗∗^	**12.2**^∗∗^	**5.1**^∗^	**4.4**^∗^	**33.8**^∗∗^	**64.7**^∗∗∗^	**50.1**^∗∗∗^
C ^∗^ Gen(S)	12	*2.5*^$^	**4.7**^∗∗^	2.1^ns^	2.1^ns^	1.0^ns^	1.6^ns^	**3.5**^∗^	1.0^ns^	1.5^ns^	**2.7**^∗^
Moisture (M)	1	**412.6**^∗∗∗^	**354.2**^∗∗∗^	**175.1**^∗∗∗^	**210.1**^∗∗∗^	**180.8**^∗∗∗^	**308.8**^∗∗∗^	**114.6**^∗∗∗^	**352.0**^∗∗∗^	**34.1**^∗∗∗^	**566.3**^∗∗∗^
M ^∗^ C	2	0.1^ns^	1.1^ns^	1.4^ns^	1.4^ns^	1.3^ns^	0.9^ns^	*3.3*^$^	**4.3**^∗^	1.3^ns^	0.4^ns^
M ^∗^ S	1	*4.1*^$^	1.0^ns^	**20.2**^∗∗^	3.5^ns^	1.6^ns^	2.8^ns^	**16.4**^∗∗^	0.2^ns^	0.2^ns^	0.7^ns^
M ^∗^ Gen(S)	6	0.8^ns^	1.3^ns^	**4.2**^∗^	2.1^ns^	**2.9**^∗^	**3.4**^∗^	**15.7**^∗∗∗^	1.2^ns^	**3.8**^∗^	1.5^ns^
M ^∗^ C ^∗^ S	2	0.2^ns^	**4.0**^∗^	0.0^ns^	1.6^ns^	2.2^ns^	2.0^ns^	1.6^ns^	1.2^ns^	0.2^ns^	1.3^ns^
M ^∗^ C ^∗^ Gen(S)	12	0.9^ns^	0.4^ns^	0.6^ns^	1.0^ns^	1.3^ns^	1.1^ns^	0.5^ns^	0.8^ns^	0.7^ns^	0.6^ns^
Block	5	**6.7**^∗^	**8.0**^∗∗∗^	**15.3**^∗∗∗^	**8.5**^∗∗∗^	**4.4**^∗∗∗^	**32.2**^∗∗∗^	**23.2**^∗∗∗^	**6.9**^∗∗∗^	**30.3**^∗∗∗^	**29.5**^∗∗∗^

*Genotypes were nested within size class. Significances are as follows: ^ns^p > 0.10; ^$^0.10 < p < 0.05; ^∗^0.01 < p < 0.05; ^∗∗^0.05 < p < 0.01; ^∗∗∗^p < 0.001. Significant values are highlighted in bold, marginally significant values in italics.*

### Consequences of Treatments for Phenotypic Characteristics

**FIGURE 3:**
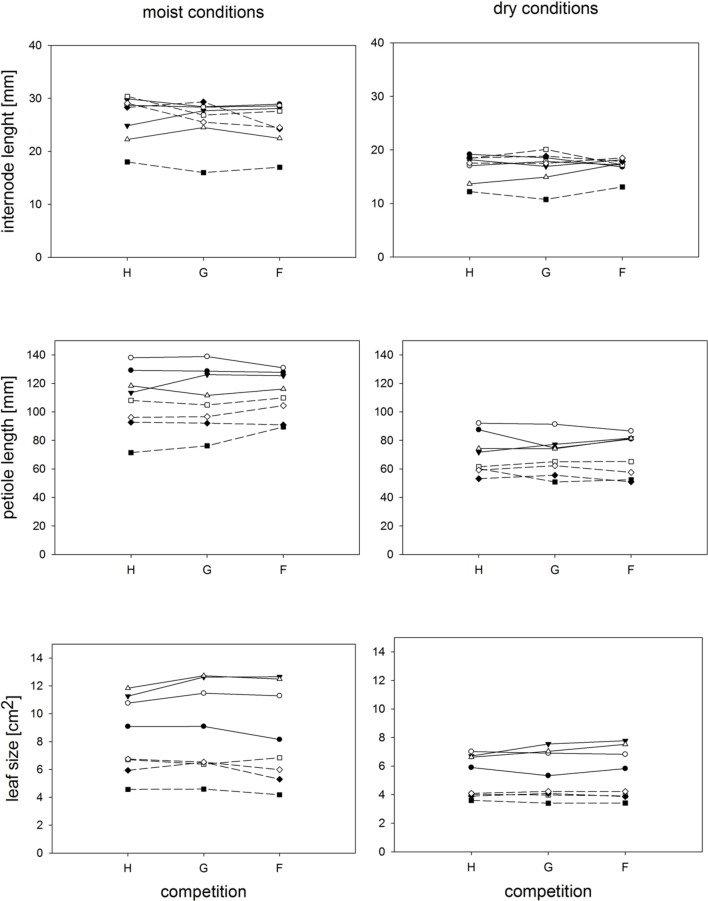
**Individual ramets: Response of parameters representing ramet architecture to competition and soil moisture treatments.** Each line represents the mean value of a specific genotype in the respective treatments. Solid lines represent large genotypes, dashed lines small genotypes. Competition type abbreviations: H: homogeneous monostands; G: phenotypically similar stands; F: phenotypically diverse stands. For significances, see **Table 2**.

**FIGURE 4:**
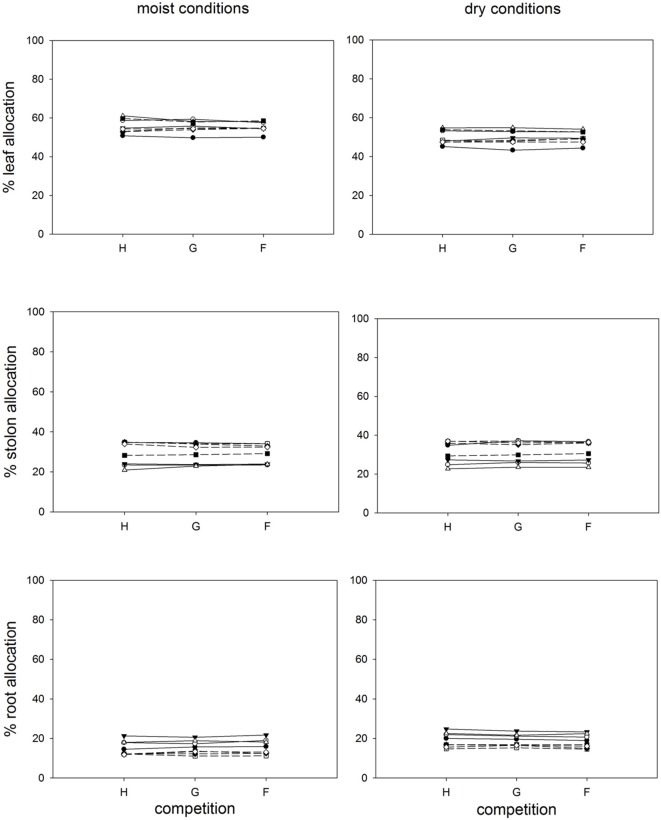
**Clonal fragments: Response of biomass allocation pattern [calculated as (g g^-1^)^∗^100] to competition and soil moisture treatments.** Each line represents the mean value of a specific genotype in the respective treatments. Solid lines represent large genotypes, dashed lines small genotypes. Competition type abbreviations: H: homogeneous monostands; G: phenotypically similar stands; F: phenotypically diverse stands. For significances, see **Table 2**.

Competition type and size class hardly affected mean ramet dry weight or ramet architecture (**Figures 2** and **3**, **Table 2**). Large-sized genotypes produced on average heavier ramets characterized by longer petioles and larger leaves. There was a very slight, but significant increase of the ramet weight of large-sized genotypes with increasing diversity, which coincided with a similarly slight decrease of ramet weight of small-sized genotypes. The relative allocation to different plant structures was fairly constant over competition treatments and size classes, with exception of allocation to roots (**Figure 4**, **Table 2**), which was on average 50% larger in large genotypes than in genotypes characterized by small ramets. This coincided with a marginally significantly reduced allocation to stolons. Across the competition treatments, the allocation pattern remained rather constant.

Soil moisture consistently affected all traits (**Figures 2–4**, **Table 2**). Plants subjected to dry conditions produced considerably less biomass, fewer and smaller ramets, and allocated more mass to roots and less to aboveground structures. Generally, the different genotypes responded similarly to soil water availability across size classes and competition treatments (**Figures 2–4**, **Table 2**). The apparently different response of ramet weight across and within size class to soil moisture was mainly due to absolute size differences and disappeared after log transformation. Within moisture conditions, ramets of large-sized genotypes remained on average twice as heavy as ramets from small-sized genotypes (moisture ^∗^ size: *F* = 4.7, *p* = 0.07; moisture ^∗^ genotype(size): *F* = 0.7, *p* = 0.6). The same held for the response of leaf size across size classes to water availability. This result was also confirmed when testing for potentially different effects of ramet size or petiole length on relative total biomass and ramet number under different soil moisture conditions within competition treatment, which revealed that there was no significant interaction between ramet size or petiole length and moisture availability (*F*-values ranging between 0.3 and 3.4).

Correspondence analysis of the whole dataset revealed that variation along the first axis corresponded very well with the separation of the genotypes according to ramet size (‘Large’ vs. ‘Small’); genotypes B7 (‘Small’) and B15 (‘Large’) in particular contributed to this correlation. Variation along axis 2 mainly corresponded with the contrast between the two water treatments (factor ‘Dry’). The third axis represented other aspects of the water treatment, with a strong correlation between allocation to roots and drought (see Supplementary Data Sheets 1 and 2). Interestingly, the treatments with different types of competition were not correlated to any of the first three axes (Pearson *r* < 0.01 in all cases), suggesting, that any effects of these treatments were unrelated to the main directions of variation in the experiment. The patterns of variation of plant traits in the two water availability treatments were remarkably similar (see Supplementary Data Sheets 1 and 2), in spite of the considerable effect of drought on the plants.

**FIGURE 5:**
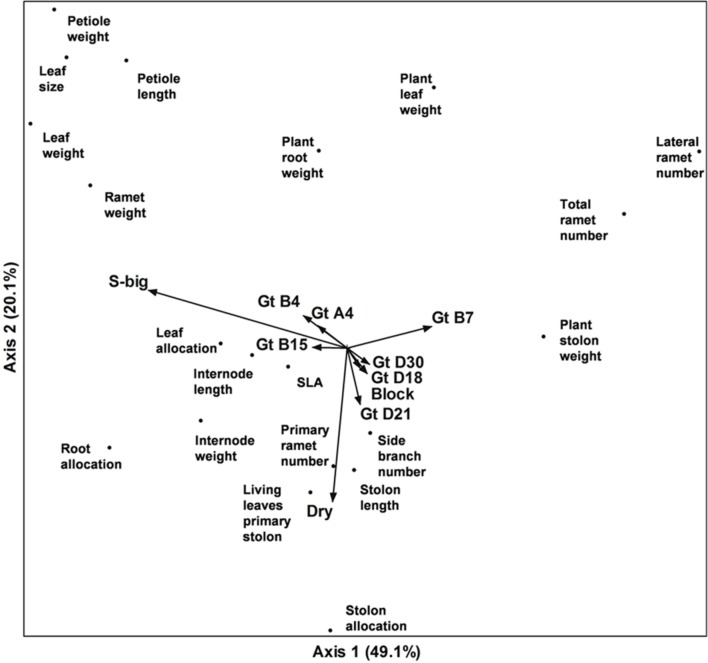
**Ordination diagram of the first two axes of a Correspondence Analysis of plant traits of eight genotypes of *Trifolium repens* with big (4) or small (4) ramets grown in three different competitive settings (within-genotype, within functional group, i.e., phenotypically similar stands, and between functional groups, i.e., phenotypically diverse stands) and with or without drought treatment.** Genotype identities, ramet size class, drought treatment, competitive setting (not visible in graph), and block were added as passive variables.

Plant traits tended to cluster in groups (**Figure 5**, Supplementary Data Sheet 2), which largely remained the same in the three analyses (moist treatment only, dry treatment only, and complete dataset – see Supplementary Data Sheet 2). One group consisted of traits measuring various aspects of individual ramets (leaf size, petiole length, and dry weight of ramet, lamina, and petiole). A second, more dispersed group consisted of traits of the whole clonal fragment such as allocation to the different organs, number of primary ramets, leaf turnover and side branches. A small third group consisted of total ramet number, lateral ramet number, and overall stolon weight. Allocation to roots was higher for genotypes with big ramets and in the drought treatment, allocation to leaves correlated strongly with the big-ramet group of plants, and allocation to stolons correlated with the drought treatment.

### Phenotypic Diversity

**FIGURE 6:**
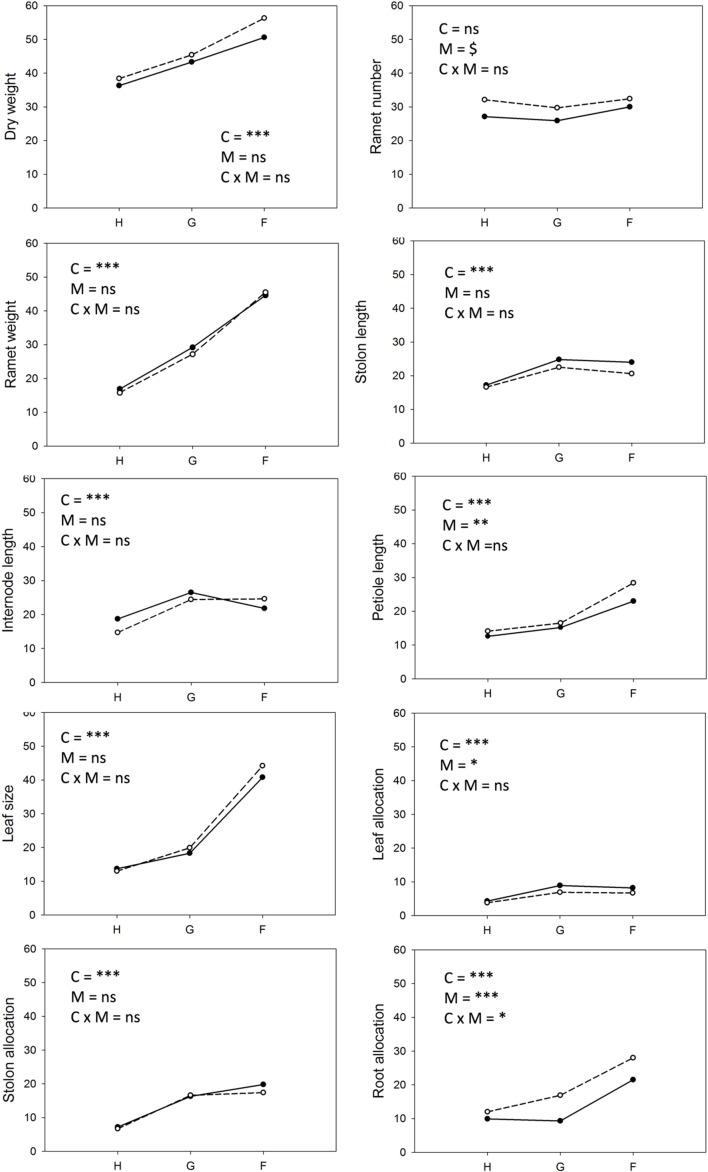
**Whole stand: Realized coefficient of variation of performance parameters, ramet and plant architectural traits and allocation pattern within stands.** Solid lines and closed symbols indicate dry conditions, dashed lines with open symbols indicate moist conditions. Competition type abbreviations: H, homogeneous monostands; G, phenotypically similar; F, phenotypically diverse. Treatment abbreviations: C, competition; M, soil moisture. Significance levels are as in **Table 2**.

Generally the realized phenotypic diversity among competing plants in the treatments with several genotypes was similar to or larger than the phenotypic diversity expressed by plants grown in monocultures. The overall variation among plants was highest for the performance parameters and lowest for biomass allocation patterns and stolon length, where the realized phenotypic variation among plants was similar to the predicted range based on the traits expressed in monocultures (**Figure 6**, Supplementary Table 2 in Supplementary Data Sheet 1). There was an almost linear increase with increasing diversity for CV of whole plant and individual ramet weight. Phenotypic, but not genetic diversity led to increased among-plant variation for leaf size and allocation to roots, while genetic and phenotypic diversity increased among-plant variation for all stolon parameters and allocation to leaves to a similar extent. Phenotypic diversity hardly affected biomass and ramet production (**Table 3**) with a few exceptions: Under moist conditions high variation in stolon length was negatively and variation in root allocation was positively associated with biomass production. Ramet number only showed negative associations with stolon allocation under dry and moist conditions.

**Table 3:** Effects of phenotypic diversity on total biomass and ramet number per tray subjected to either moist or dry conditions.

	Biomass		Ramet number	
	Moist	Dry	Moist	Dry
Model	**2.86**^∗∗^	1.16^ns^	0.83^ns^	1.29^ns^
Ramet weight	0.15^ns^	–0.13^ns^	–0.04^ns^	*0.34*^$^
Stolon length	**–0.32**^∗∗^	–0.06^ns^	0.06^ns^	0.13^ns^
Internode length	–0.07^ns^	–0.12^ns^	–0.06^ns^	–0.19^ns^
Petiole length	–0.15^ns^	–0.11^ns^	0.05^ns^	0.09^ns^
Leaf size	–0.27^ns^	0.14^ns^	0.10^ns^	–*0.31*^$^
Root allocation	**0.30**^∗^	0.16^ns^	–0.06^ns^	0.16^ns^
Stolon allocation	0.08^ns^	–0.17^ns^	–*0.35*^$^	**–0.44**^∗^
Leaf allocation	0.09^ns^	0.27^ns^	0.12^ns^	0.18^ns^

*A positive regression coefficient indicates that high variability of a specific traits is associated to higher biomass or ramet number, a negative coefficient indicates that high variability of a trait is associated to relatively lower biomass or ramet number. Data were analyzed by means of multiple regression. For the overall model the F-value, for the individual traits the standardized regression coefficients and their significance are given. Significances are as follows: ^ns^p > 0.10; ^$^0.10 < p < 0.05; ^∗^0.01 < p < 0.05; ^∗∗^0.05 < p < 0.01; ^∗∗∗^p < 0.001. Significant values are highlighted in bold, marginally significant values in italics.*

### Stand Performance

**FIGURE 7:**
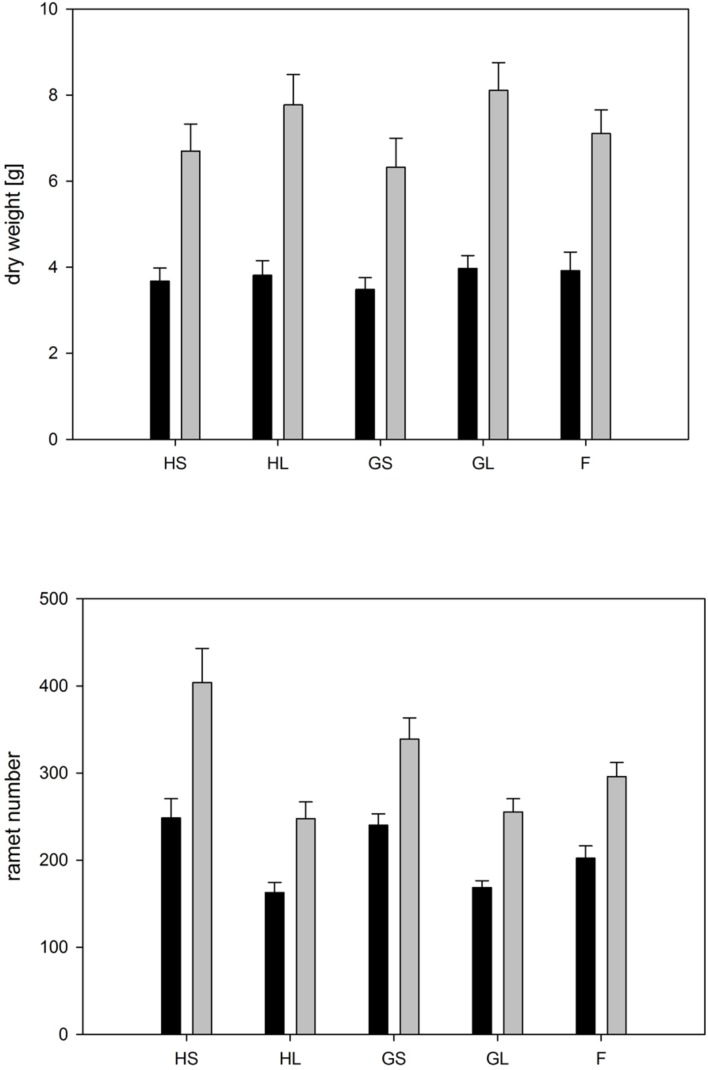
**Whole stand: Overall dry weight and ramet number per tray for each competition treatment and water availability.** Dark bars represent trays subjected to dry conditions, light bars trays subjected to moist conditions. Treatment abbreviations are as follows: H: homogeneous monostands; G: phenotypically similar stands consisting of four different genotypes with similar phenotype; F: phenotypically diverse stands consisting of two large and two small genotypes; L: genotypes with large ramets; S: genotypes with small ramets. For significances, see **Table 4**.

The type of competition significantly affected overall ramet and biomass production by the whole stand (**Figure 7**, **Table 4**). If grown in monocultures stands consisting of large genotypes produced significantly fewer ramets and a higher total biomass than monocultures consisting of small genotypes. This result was similar for phenotypically similar stands consisting of the four genotypes characterized by large ramets. Overall, low soil moisture availability significantly reduced overall biomass by up to 50% and ramet number by up to 25% (**Figure 7**, **Table 4**). The negative effect of low moisture availability on ramet number depended on the type of competition. Monocultures consisting of small sized genotypes responded most strongly to the drought treatments, while phenotypically diverse stands showed the weakest response to drought treatments.

**Table 4:** Results of a two-way ANOVA testing for the effects of competition treatment and soil moisture on overall production parameters per tray.

	df	Overall biomass per tray	Overall ramet number per tray
Competition	4	**4.5**^∗∗^	**49.2**^∗∗∗^
Soil moisture	1	**383.3**^∗∗∗^	**206.6**^∗∗∗^
Competition ^∗^ moisture	4	1.9^ns^	**4.4**^∗∗^
Block	5	**9.9**^∗∗∗^	**8.8**^∗∗∗^

*F-values and their significance are given. Significances are as follows: ^ns^p > 0.10; ^$^0.10 < p < 0.05; ^∗^0.01 < p < 0.05; ^∗∗^0.05 < p < 0.01; ^∗∗∗^p < 0.001. Significant values are highlighted in bold, marginally significant values in italics*.

No evidence of overyielding of the mixtures was found. The statistical analysis (see **Table 5**) indicated that the net diversity effect did not significantly differ from 0. This was true for both, populations subjected to different competition treatments and to different soil moisture availability. In addition, the variation among traits hardly explained variation in biomass and ramet production (**Table 3**). Across competition treatments variation in the different traits explained only total biomass in stands subjected to moist conditions (**Table 3**). Under these conditions high variation in stolon length was negatively associated with overall biomass production, while high variation in allocation to roots was positively associated with overall biomass production.

**Table 5:** Results of a two-way ANOVA testing for the effects of competition treatment and soil moisture on the increase in total mass in the mixtures compared to the expected yield from the monocultures (net diversity effect, Δy) and its components, the complementarity effect (CE) and the selection effect (SE).

	df	Δy	CE	SE
Intercept	1	0.05^ns^	0.01^ns^	0.01^ns^
Competition	2	2.39^ns^	2.14^ns^	0.11^ns^
Soil moisture	1	1.47^ns^	0.02^ns^	0.00^ns^
Competition ^∗^ moisture	2	0.55^ns^	0.50^ns^	0.66^ns^
block	5	**2.72**^∗^	**4.64**^∗∗^	**2.92**^∗^

*F-values and their significance are given. Significances are as follows: ^ns^p > 0.10; ^$^0.10 < p < 0.05; ^∗^0.01 < p < 0.05; ^∗∗^0.05 < p < 0.01; ^∗∗∗^p < 0.001. Significant values are highlighted in bold, marginally significant values in italics*.

## Discussion

Our results clearly show that the relative performance of phenotypically different genotypes was neither affected by the competitive environment nor by the prevalent environmental conditions, resulting in an almost constant ranking among genotypes. They further show that highly diverse populations did not outperform monocultures in terms of increased ramet or biomass production or increased resistance to environmental fluctuations. However, the increased variation in biomass production and decreased variation in ramet production with increasing diversity showed that these competition treatments affected genotypes differently. This latter pattern was consistent across different levels of water availability. These results may be explained by a highly conservative, evolutionarily fixed, set of character combinations. Generally evolutionary changes and genotypic diversity are not independent. Increased variation among genotypes creates a higher potential for selection to act upon, and will also eventually result in faster evolutionary changes. Based on the results from the present experiment, diversity can be hypothesized to be lost at different speeds depending on the main trait under selection, i.e., biomass or ramet number; but this loss of genetic diversity appears to be largely unaffected by water availability. These results are in contrast to the common expectation that diversity increases resistance to environmental change and that environmental variation can lead to a maintenance of genetic diversity in populations. Below, we discuss our results in the light of community productivity and resilience.

### Relative Genotype Performance Does Not Change across Water Treatments

Contrary to our expectation, the relative performance of genotypes was not affected by water availability. Our expectation was based on the idea that in highly competitive herbaceous communities the performance of plants strongly depends on the relative positioning of leaves within the canopy (Anten and Hirose, 1998; Anten et al., 2005), leading to a competitive advantage of taller individuals over shorter ones (Weiner, 1985, 1990). As *T. repens* had formed dense canopies throughout the experiment, resulting in an up to fivefold elongation of the vertically oriented petioles in moist conditions, we had expected genotypes with inherently larger ramets to outperform genotypes with inherently smaller ramets if subjected to moist conditions, but not under dry conditions where canopy density was lower and the light gradient less steep. Interestingly, even though plants responded to moisture treatments with altering petiole elongation, the relative positioning of the leaves within the canopy remained constant across moisture treatments, as petiole elongation of small and large ramets was similarly reduced by the drought treatment, and thus the relative height distribution across genotypes did not change much. This constant distribution of leaves of different genotypes may explain why, even though the leaf area index was lower under drought treatments, no shift in genotype ranking took place despite the large differences in standing crop biomass and why there was no shift in genotypic and phenotypic selection across different drought levels.

The riverine populations of *T. repens* from which the genotypes used in this experiment originated were characterized by a 97% within-population molecular variance as compared to a 3% among-population variance (J.L. Peters, unpublished results). This is in line with results on other clonal plant species characterized by high within-population variation exceeding among-population genetic variation (Huber et al., 2014). Spatiotemporal environmental heterogeneity has been argued to be an important driver explaining the high genetic variation in many clonal plant populations, as environmental perturbations, with slight advantages or disadvantages for given genotypes, can be expected to reduce the likelihood of potential genotypic variation loss. The proliferation of well-adapted genotypes and associated loss of less well-adapted genotypes can result in a decreased genetic variation over time (Stuefer et al., 2009; Gladieux et al., 2015). This, however, is not supported by the results of our experiment: genotype ranking remained relatively constant across environmental conditions, despite the strongly altered growth and phenotype in dry as compared to moist conditions.

Maintenance of genetic variation is thought to be important for community stability and resilience to environmental change. However, proliferation of well-adapted genotypes and associated loss of less well-adapted genotypes can result in a decreased genetic variation over time (Stuefer et al., 2009; Gladieux et al., 2015). In our experiment, the different genotypes were characterized by different performance across competition treatments, which is likely to result in loss of genotypic variation, especially as the same genotypes were favored across environmental conditions. This may indicate a potentially high short term resistance of population structure in response to environmental variation in *T. repens*. In a similar, long-term experiment with the clonal species *P. reptans* it has been found that over time the community became dominated by a few genotypes while other genotypes completely disappeared (Stuefer et al., 2009). In concert these results imply that while genetic diversity can be lost in stable conditions on the long run, environmental variation does not have to lead to maintenance of genetic diversity, as different environmental conditions do not necessarily favor different genotypes.

### Diversity Does Not Affect Overall Population Performance

In our experiment, overall yield was not affected by genotypic or phenotypic diversity. This is in contrast to the hypothesis that genetically and phenotypically diverse stands would have a higher overall performance and be more persistent (Grimm and Wissel, 1997) to drought as different genotypes may occupy different ecological niches. Several studies have shown that in multispecies communities functional diversity of stands positively affects community growth and stability (van Ruijven and Berendse, 2005; Gross et al., 2014; Brotherton and Joyce, 2015; Venail et al., 2015), which has been attributed to various mechanisms such as niche differentiation, positive effects of high diversity of associated soil and herbivore communities, and the lower accumulation of species specific soil pathogen loads (de Kroon et al., 2012; Hendriks et al., 2013; Bardgett and van der Putten, 2014). Within-species genetic diversity has been shown to have similar positive effects on population growth and stability to environmental variation both, for plant and insect populations (Reusch et al., 2005; Ellers et al., 2011; Drummond and Vellend, 2012; Hughes, 2014). The positive effects of genetic diversity on productivity have been attributed to niche complementarity among phenotypically diverse genotypes. While genetic diversity has led to increased community performance in some cases, our experiment supports other studies performed under controlled and (semi)natural conditions where no such positive effect was found (Vellend et al., 2010; Tomimatsu et al., 2014; Williams et al., 2014). In line with the predictions of Drummond and Vellend (2012) large-sized genotypes in our experiment did produce a higher total biomass if grown in monocultures or in competition with other large-sized genotypes, while small-sized genotypes performed better in terms of ramet number if grown in monoculture or in communities consisting of small sized genotypes. However, this difference did not lead to a relative overyielding in terms of, respectively, biomass or ramet number if plants were grown in phenotypically diverse communities. While genotypes did maintain their relative position across treatments, overall productivity was not higher in phenotypically diverse stands than predicted from the average performance in homogeneous stands. This indicates that complementarity and increased productivity may not be the main mechanism favoring genetically diverse stands in stoloniferous grassland species like *T. repens*.

### Relative Performance Depends on Whether Biomass Increment or Ramet Number is under Selection

Interestingly, there was a much higher variation in performance among genotypes in phenotypically diverse stands if performance was expressed in terms of dry weight, than if expressed in terms of ramet number. This was conspicuously different from the genotype performance predicted by growth in monocultures, which were characterized by a relatively constant biomass, but highly variable ramet number. Different genotypes had different mechanisms to reach the same biomass production, either by producing many small or few large ramets, a typical pattern for organisms characterized by size-number trade-offs (Huber and Wiggerman, 1997; Vermeulen and During, 2010). However, in phenotypically diverse populations the relative advantage of genotypes with large ramets over genotypes with small ramets led to a shift in the rate of ramet production, resulting in a convergence of ramet production rate across phenotypes. Depending on the prevalent conditions, either high reproduction or high biomass may be favored by selection. Producing many ramets may be favorable in highly disturbed environments, where ramets may serve as a bud bank, enabling plants to regrow after part of the vegetation has been removed. In relatively stable, highly competitive environments genotypes with large ramets maintaining high biomass production may be characterized by higher performance which might lead to a loss of smaller sized genotypes over time, even if they have a potentially higher ramet production rate (Stuefer et al., 2009; Vermeulen et al., 2013). Testing this hypothesis would require to subject genotypes from different positions along the ramet number-ramet size trade-offs gradient to selection regimes differing in disturbance and plant density.

### *Trifolium repens* is Characterized by Constant Character Combinations

While the phenotypes of a given genotype largely remained similar across competition treatments, drought treatments induced conspicuously different phenotypes. However, the relationship among the different traits remained surprisingly constant over drought treatments. Selection driven by heterogeneous conditions would allow for genotypes to co-exist if the rank order of performance varies under different conditions, or if genotypes show different plastic alterations of traits associated with increased performance. In our experiment, neither the relative performance of genotypes was different among environments, nor were the within-genotype trait relations changed. In addition, even across the different competition treatments the trait values remained relatively constant, indicating a highly integrated phenotype, where most traits appear to respond in concert. This is also in line with the rigid structure of this species, which results in limited opportunities for changed growth and allocation pattern. This rigidity in character combination may explain the lack of shift in genotypes across environmental conditions as well as the extraordinarily high within population genetic variation in natural populations, as there may be little potential for selection on genotypes with a specific, highly favorable, sets of traits.

## Conclusion

A clear pattern emerges from our experiment. In contrast to our hypotheses, genotype size and architecture neither affected the overall ranking of the different genotypes in response to the different competitive environments, nor in response to water availability. These results were consistently supported across the extensive set of different analyses: as can be seen by the lack of a significant association between type of competition and the first two axes in the CA, as well as the absence of over-yielding, which would indicate a difference in relative genotype performance under a given set of environmental conditions. These results may indicate that increased frequency of drought spells, as predicted by global change scenarios, will not necessarily lead to immediate shifts in genotype abundance of this important pasture legume. However, the genotypes did differ in their relative performance, which implies that some genotypes may disappear in the long run, a process which does not seem to be affected by drought. The speed of this process is likely to depend on competitive environments, as the relative difference in fitness expressed by the eight genotypes did vary across competition environments. Depending on the main trait under selection, overall biomass production or ramet number, the long term changes may take at a different speed. The convergence of ramet number in diverse mixtures implies that diversity can be maintained if ramet number is under selection. Alternatively if total biomass is the main trait under selection, genotypes may get lost on the long run. However, the eventual loss in genetic diversity may not be associated to immediate negative consequences. This is supported by our results (i.e., the lack of correlation between diversity and overall stand performance) which show that phenotypic and genetic diversity are unlikely to contribute to increased productivity or increased resistance to drought in stands dominated by white clover. These results suggest that communities dominated by *T. repens* may be relatively resilient in changing environments characterized by higher frequency of drought events.

## Author Contributions

This paper is the result of a close cooperation of the authors with significant contribution of all authors in different parts of the process. HH was involved in all stages of the experiment, including planning, analyses and writing of the manuscript. FB performed the experiment and the initial analyses of the data and wrote a master thesis on which this paper is based. HD, NA, and PV were involved in conceptual interpretation of the data, data analyses, and writing of the manuscript.

## Conflict of Interest Statement

The authors declare that the research was conducted in the absence of any commercial or financial relationships that could be construed as a potential conflict of interest.
