# Supplementary material for: Genotypic and Phenotypic Diversity Does Not Affect Productivity and Drought Response in Competitive Stands of Trifolium repens
Source: Front Plant Sci. 2016 Mar 29;7:364. doi: 10.3389/fpls.2016.00364 (PMC4809891; doi:10.3389/fpls.2016.00364)
Supplement: Supplementary file 1 [file Data_Sheet_1.PDF]

Supplementary Material

Does genotypic and phenotypic diversity affect drought response in Trifolium repens?

Heidrun Huber*, Heinjo J. During, Fabienne Bruine de Bruin, Peter Vermeulen & Niels P.R. Anten

*** Correspondence:** Corresponding Author: h.huber@science.ru.nl

1 Supplementary Figures and Tables

1.1 Supplementary Figures

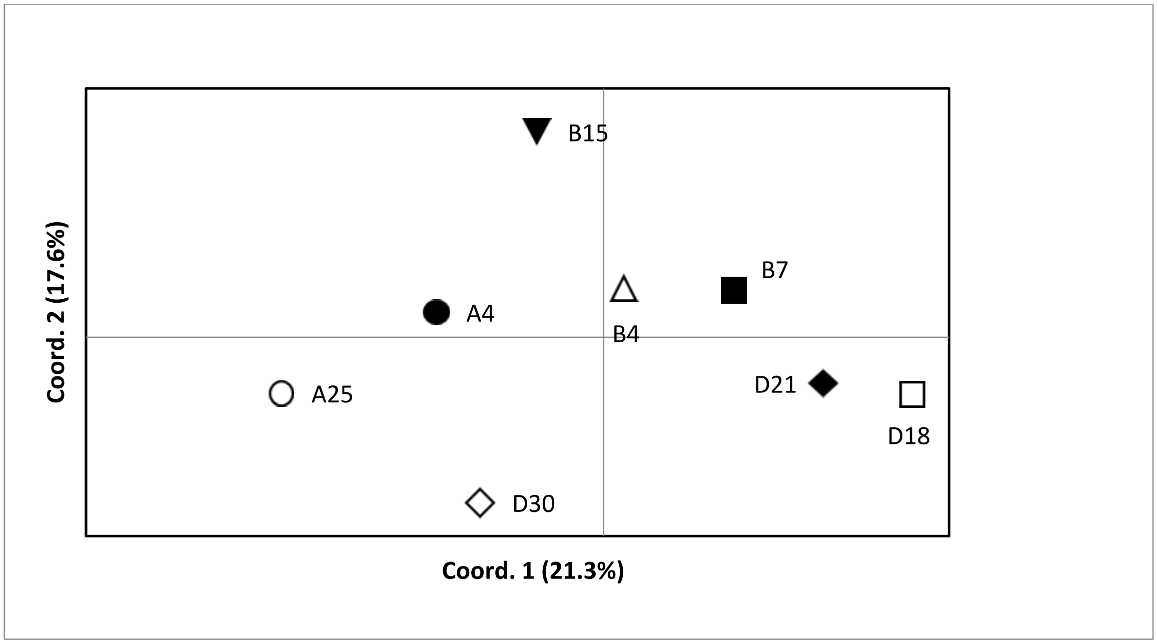

Supplementary Figure 1: PCoA coordinates of the eight genotypes used in the experiment. The PCoA coordinates are based on 2 primer combinations resulting in a total of 129 markers. The first two coordinates and the % variation explained by the two axes is given.

1.2 Supplementary Tables

Supplementary Table 1: Values of correlations (r, r^2 and Kendall's tau) between the supplementary traits of the experimental plants and the first three axes of the Correspondence Analysis of the full dataset (N=737).

Axis:	1			2			3		
	r	r-sq	tau	r	r-sq	tau	r	r-sq	tau
S-big	-.582	.338	-.502	.323	.104	.272	-.102	.010	-.070
Dry	-.158	.025	-.129	-.529	.280	-.439	.543	.295	.475
comp H	.041	.002	.017	.001	.000	-.005	-.022	.000	-.015
comp G	-.023	.001	-.009	-.014	.000	-.012	-.010	.000	-.013
comp F	-.024	.001	-.011	.013	.000	.017	.035	.001	.031
Gt A25	-.138	.019	-.106	.010	.000	.005	-.173	.030	-.147
Gt A4	-.226	.051	-.209	.202	.041	.174	-.100	.010	-.073
Gt B15	-.244	.060	-.215	.046	.002	.031	-.073	.005	-.038
Gt B4	-.276	.076	-.232	.230	.053	.201	.187	.035	.148
Gt B7	.379	.144	.276	.172	.029	.144	.522	.272	.384
Gt D18	.151	.023	.143	-.182	.033	-.153	-.167	.028	-.134
Gt D21	.148	.022	.152	-.306	.094	-.244	-.129	.017	-.095
Gt D30	.195	.038	.182	-.165	.027	-.153	-.069	.005	-.047
Block	.183	.033	.158	-.206	.042	-.147	.014	.000	.005

Supplementary Table 2: Potential variation among genotypes calculated as the CV among genotypes growing in monocultures

	Dry weight	Ramet number	Ramet weight	Stolon length	Inter-node length	Petiole length	Leaf size	Root allo-cation	Stolon allo-cation	Leaf allo-cation
Wet conditions										
Overall	13.03	27.34	30.68	15.16	15.51	18.43	30.60	22.53	18.89	6.22
Large genotypes	4.71	11.42	8.06	10.92	11.57	7.65	9.56	13.30	20.53	6.95
Small genotypes	15.75	13.14	25.86	16.19	18.64	14.33	14.71	1.34	8.36	5.05
Dry conditions										
Overall	9.45	24.96	27.94	15.30	14.01	18.79	26.15	17.90	17.28	6.78
Large genotypes	6.32	9.93	10.40	13.34	12.19	10.55	6.21	7.57	16.69	7.75
Small genotypes	11.53	16.07	20.23	12.29	15.61	5.47	4.86	5.52	9.06	5.40
