# Supplementary material for: Genotypic and Phenotypic Diversity Does Not Affect Productivity and Drought Response in Competitive Stands of Trifolium repens
Source: Front Plant Sci. 2016 Mar 29;7:364. doi: 10.3389/fpls.2016.00364 (PMC4809891; doi:10.3389/fpls.2016.00364)
Supplement: Supplementary file 2 [file Data_Sheet_2.PDF]

Supplementary Material

Does genotypic and phenotypic diversity affect drought response in Trifolium repens

Heidrun Huber*, Heinjo J. During, Fabienne Bruine de Bruin, Peter Vermeulen & Niels P.R. Anten

*** Correspondence:** Corresponding Author: h.huber@science.ru.nl

1 Supplementary Text

Correspondence analyses

Our experiment involved plants of *Trifolium repens* with 21 traits, belonging to eight genotypes divided into two size groups according to average ramet size (Big/Small), three types of competition with increasing degree of genetic variation among competitors (within-genotype, within-size group and between size-group), and two water availability treatments. In order to get an overview of the major axes of variation in plant traits and the effects of type of competition and water availability, we used Correspondence Analysis as implemented in PC-Ord 6.03 (option RA; McCune and Mefford 2011). We analyzed the extensive datasets of the measured traits, including more traits then presented in the main manuscript but excluding the composite trait total plant weight in both water treatments separately and in combination, with the major directions of variation correlated to treatments, block effect, type of competition and individual genotypes calculated afterwards and added as supplementary variables in the ordination graphs. Here we present the results of these analyses in more detail.

For both water treatments separately and for the full dataset we present ordination diagrams of the morphological plant traits in the planes of the axes 1 & 2 and 1 & 3, respectively. The red arrows represent the passive correlations of the experimental traits: size group, competition type, water treatment and block effect, with the ordination axes. Each visual presentation is followed by a table showing the correlations of these experimental traits with the ordination axes.

In all three analyses, the amount of variation explained by the first three axes is very large: 86-87%. The morphological plant traits themselves are mutually strongly correlated and form clusters, which are largely similar in all three analyses. Consequently, the results of the three ordinations look quite similar (apart from mirroring effects which often occur in this type of analysis). One cluster contains traits associated with ramet size (dry weight of ramet, lamina, and petiole; lamina area; petiole length). Unsurprisingly, higher values of these traits are correlated with the factor S-Big. Dry weight and length of individual ramet internodes have a deviating position in all graphs, but these show also higher values for plants belonging to genotypes of the Big group. The three types of competition are hardly correlated with any of the first three axes in all three analyses (see the tables below the graphs), demonstrating that competitive effects on the plants hardly depended on the level of genetic diversity in the arenas.

In both water availability treatments, the first axis (explaining over 50% of the variance) strongly correlates with the two size groups of genotypes. Important traits determining this axis are number of ramets (lower in big-leaf genotypes) against dry weight of ramets and ramet parts. The second axis (explaining ca 20% of the variance) is related to variation between genotypes within the two groups, with a rather deviating position of the small-leaved genotype B7. Along this axis dry weight and length of the main stolon stand opposite to the number of ramets, SLA, and percentage allocation to leaves. The main contrast along the third axis is connected with dry weights of plant parts (particularly roots) on the one hand and allocation parameters and SLA on the other hand. In the moist treatment, factor Block is correlated with axes one and two and related with plant size, with larger plants but slightly smaller ramets in later blocks. In the dry treatment Block is correlated with axis three, and is linked to ramet size.

The analysis of the full dataset with plants from both water availability treatments shows, that the clusters of plant traits are still largely the same. Axis one is still correlated with the division of genotypes into the two size groups, but the second and third axes are now strongly correlated with the water availability treatment. Drought seems to lead to a higher allocation of mass to stolons and roots and lower dry weights of plant parts. Apparently, the water treatments did have a strong effect on the plants, but the relations between and traits of the different plant parts are highly conserved. Drought mainly affected investment in roots and lead to smaller plants and individual ramets.

Reference

McCune, B & Mefford, MJ. 2011. PC-ORD 6. 03 Multivariate Analysis of Ecological Data. MjM Software Design, Gleneden Beach, OR, USA

2 Supplementary Figures and Tables

2.1 Supplementary Figures

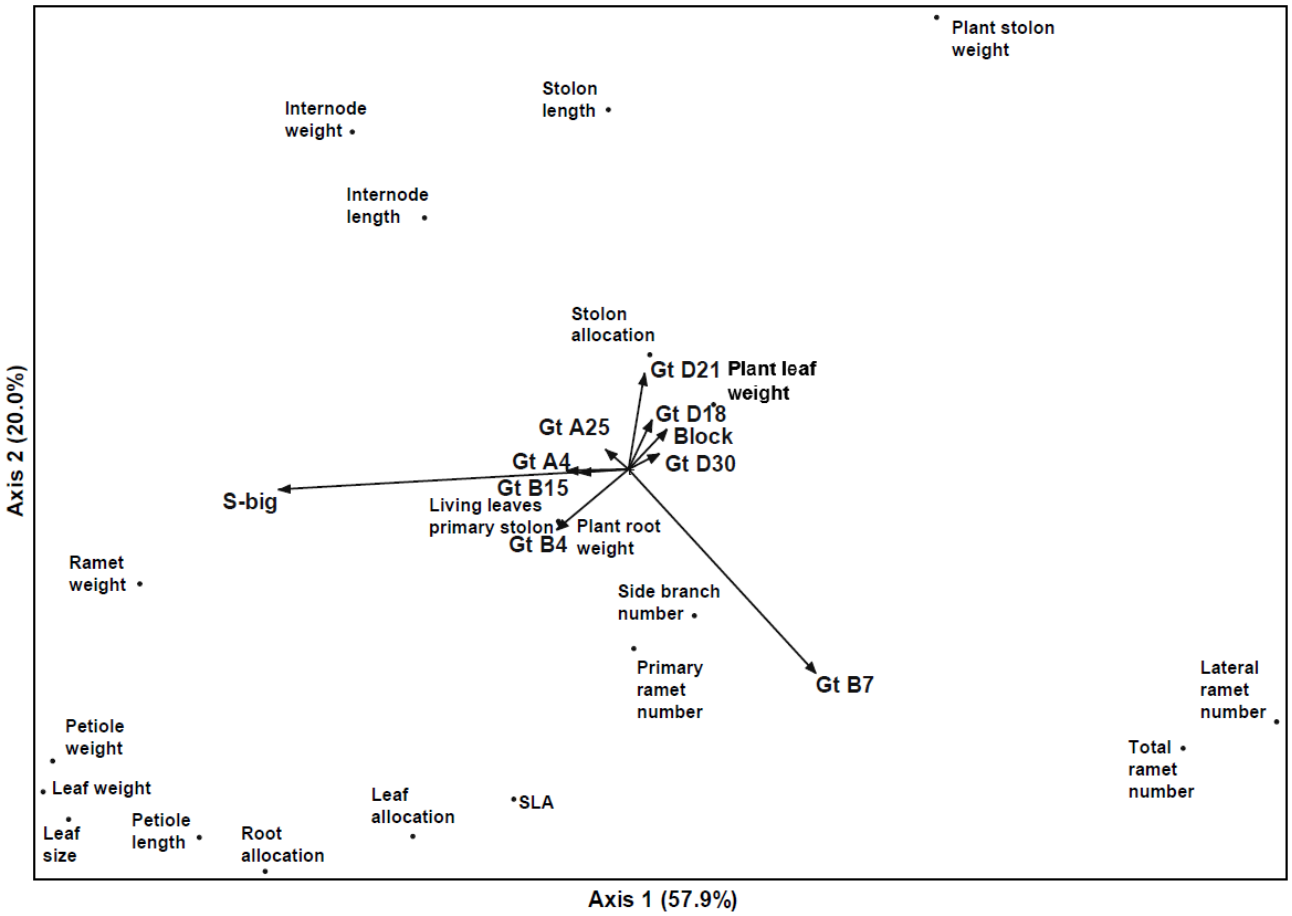

Supplementary file Figure S-1. Ordination diagram of the first two axes of a Correspondence Analysis of plant traits of eight genotypes of *Trifolium repens* with big (4) or small (4) ramets grown in three different competitive settings (within-genotype, within functional group, and between functional groups), well-watered treatment. Genotype identities, ramet size class, competitive setting (not visible in graph) and block added as passive variables.

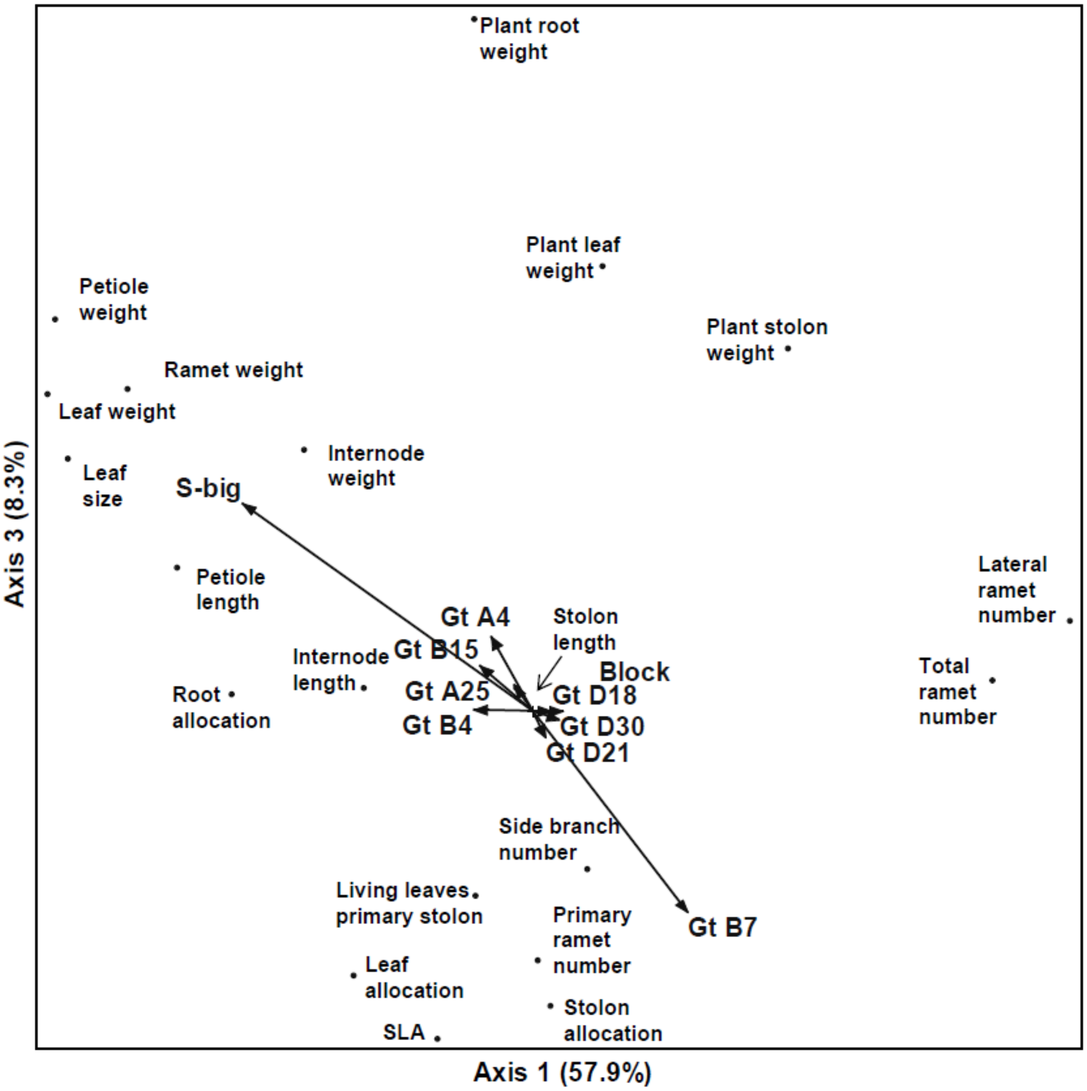

Supplementary file Figure S-2. Ordination diagram of axes 1 and 3 of a Correspondence Analysis of plant traits of eight genotypes of *Trifolium repens* with big (4) or small (4) ramets grown in three different competitive settings (within-genotype, within functional group, and between functional groups), well-watered treatment. Genotype identities, ramet size class, competitive setting (not visible in graph) and block added as passive variables.

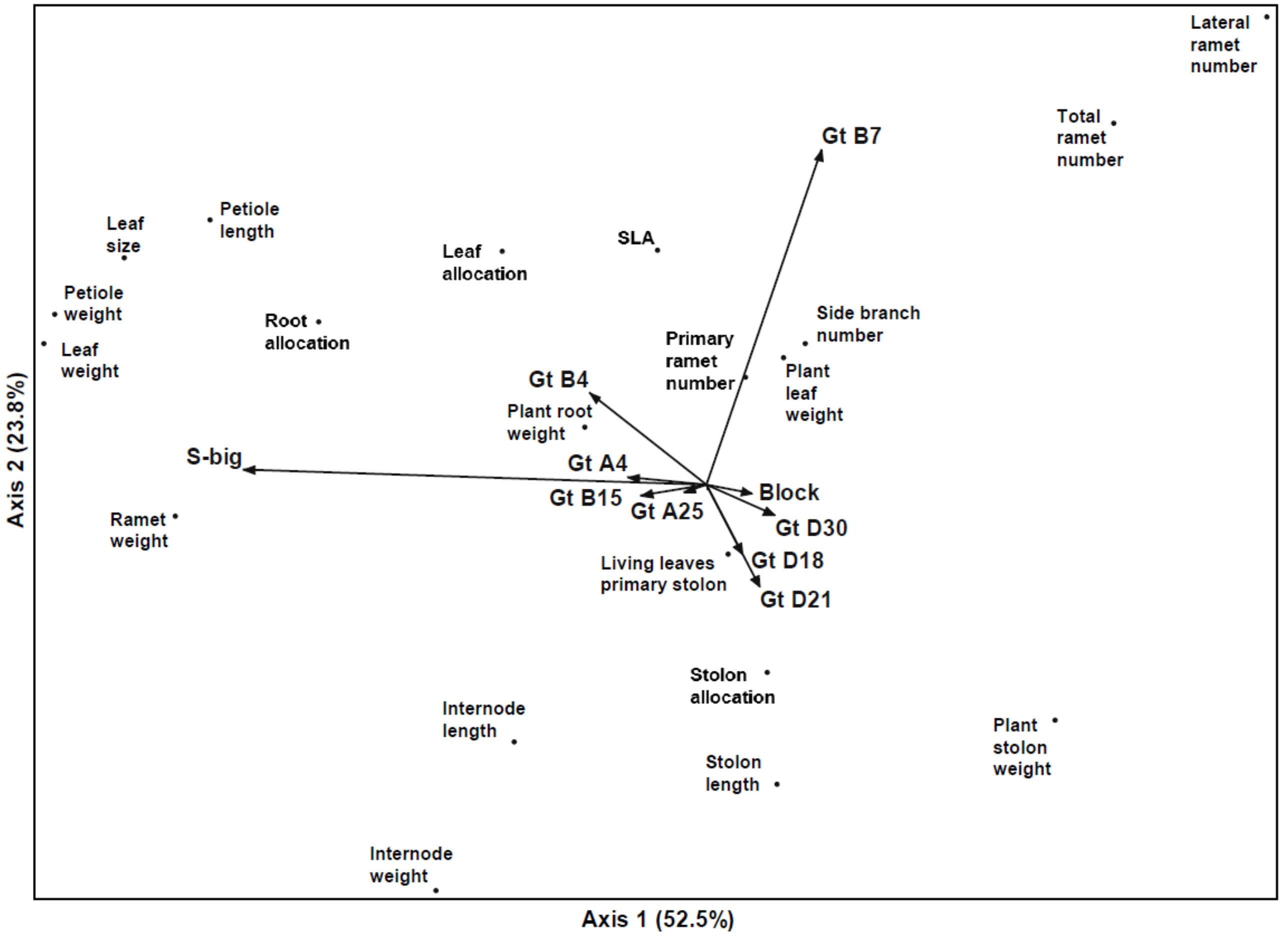

Supplementary file Figure S-3. Ordination diagram of the first two axes of a Correspondence Analysis of plant traits of eight genotypes of *Trifolium repens* with big (4) or small (4) ramets grown in three different competitive settings (within-genotype, within functional group, and between functional groups), drought treatment. Genotype identities, ramet size class, competitive setting (not visible in graph) and block added as passive variables.

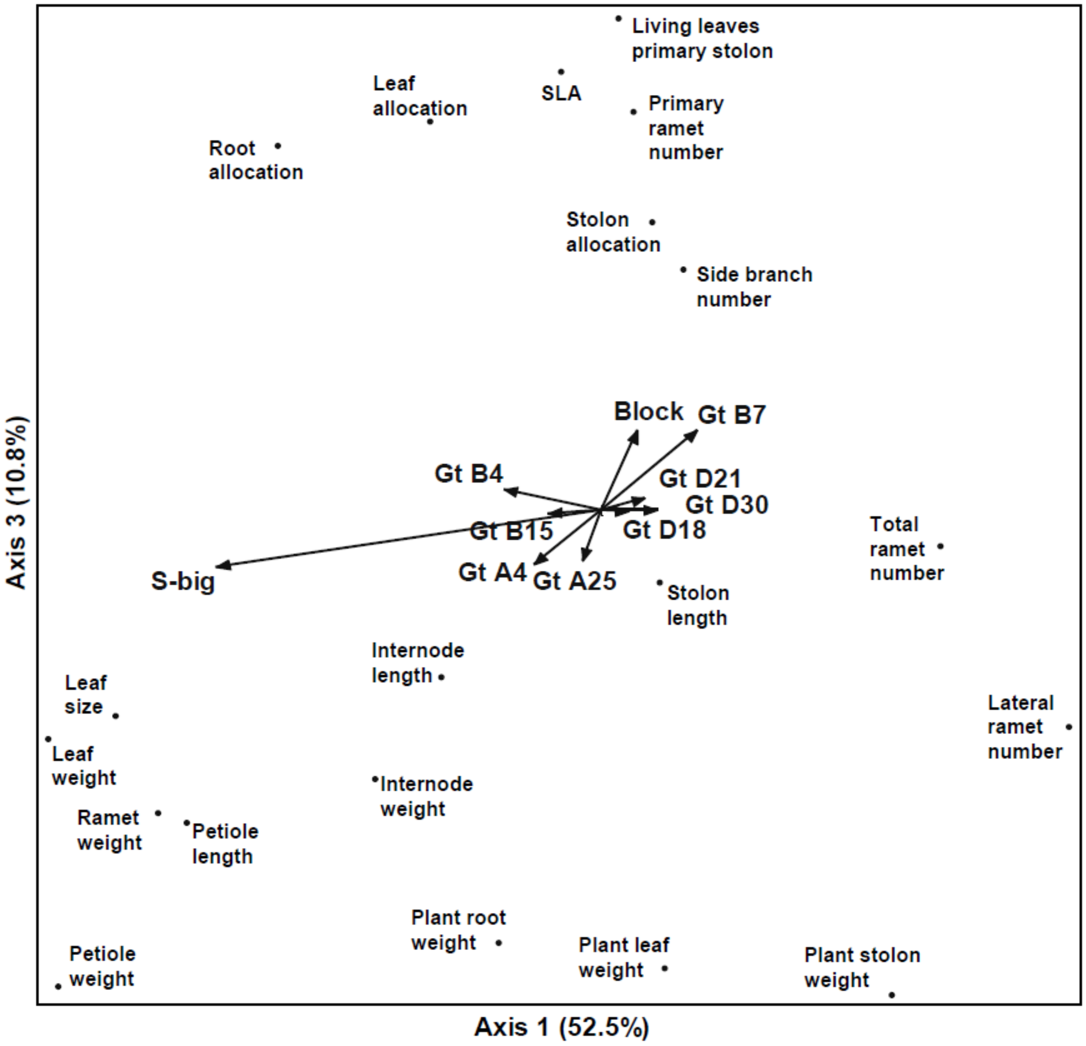

Supplementary file Figure S-4. Ordination diagram of axes 1 and 3 of a Correspondence Analysis of plant traits of eight genotypes of *Trifolium repens* with big (4) or small (4) ramets grown in three different competitive settings (within-genotype, within functional group, and between functional groups), drought treatment. Genotype identities, ramet size class, competitive setting (not visible in graph) and block added as passive variables.

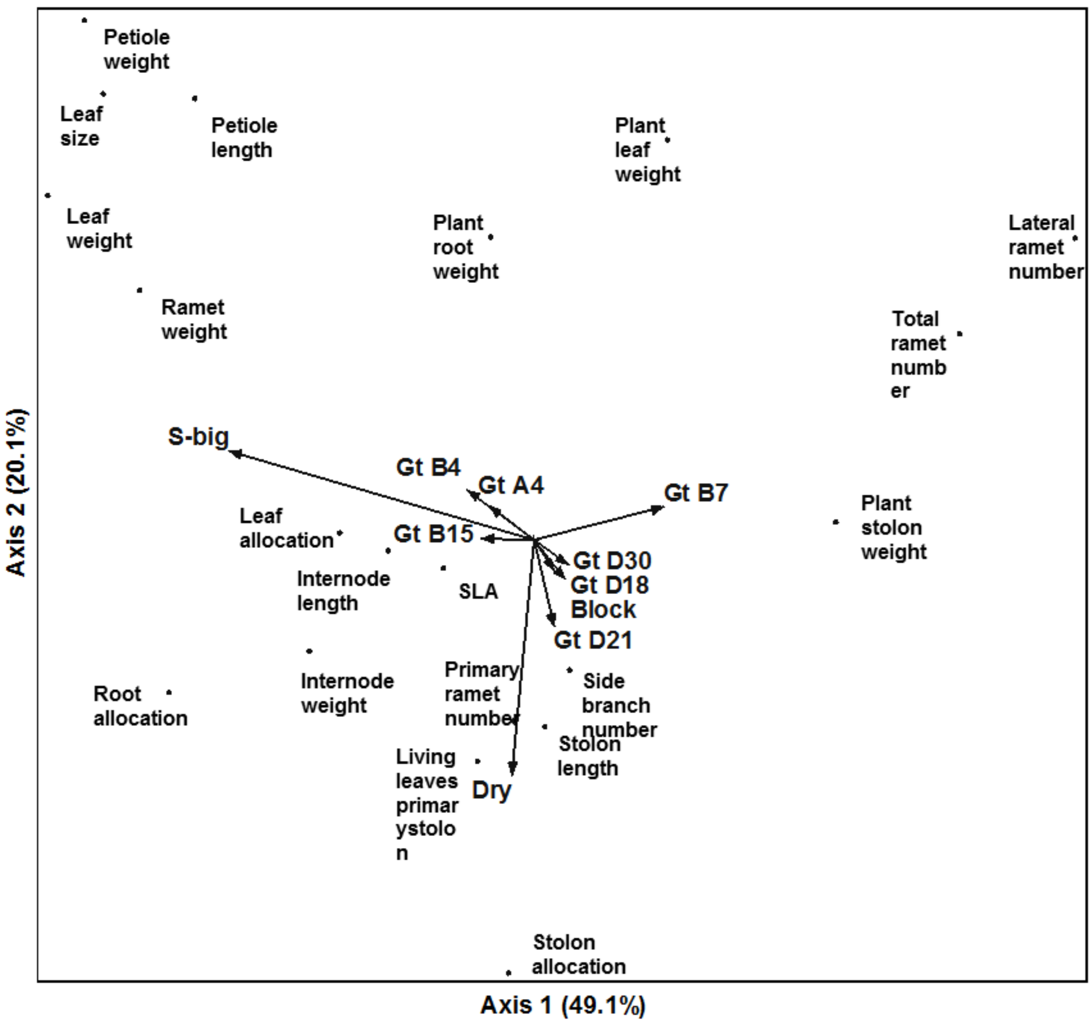

Supplementary file Figure S-5. Ordination diagram of the first two axes of a Correspondence Analysis of plant traits of eight genotypes of *Trifolium repens* with big (4) or small (4) ramets grown in three different competitive settings (within-genotype, within functional group, and between functional groups), all water treatments. Genotype identities, ramet size class, drought treatment, competitive setting (not visible in graph) and block added as passive variables.

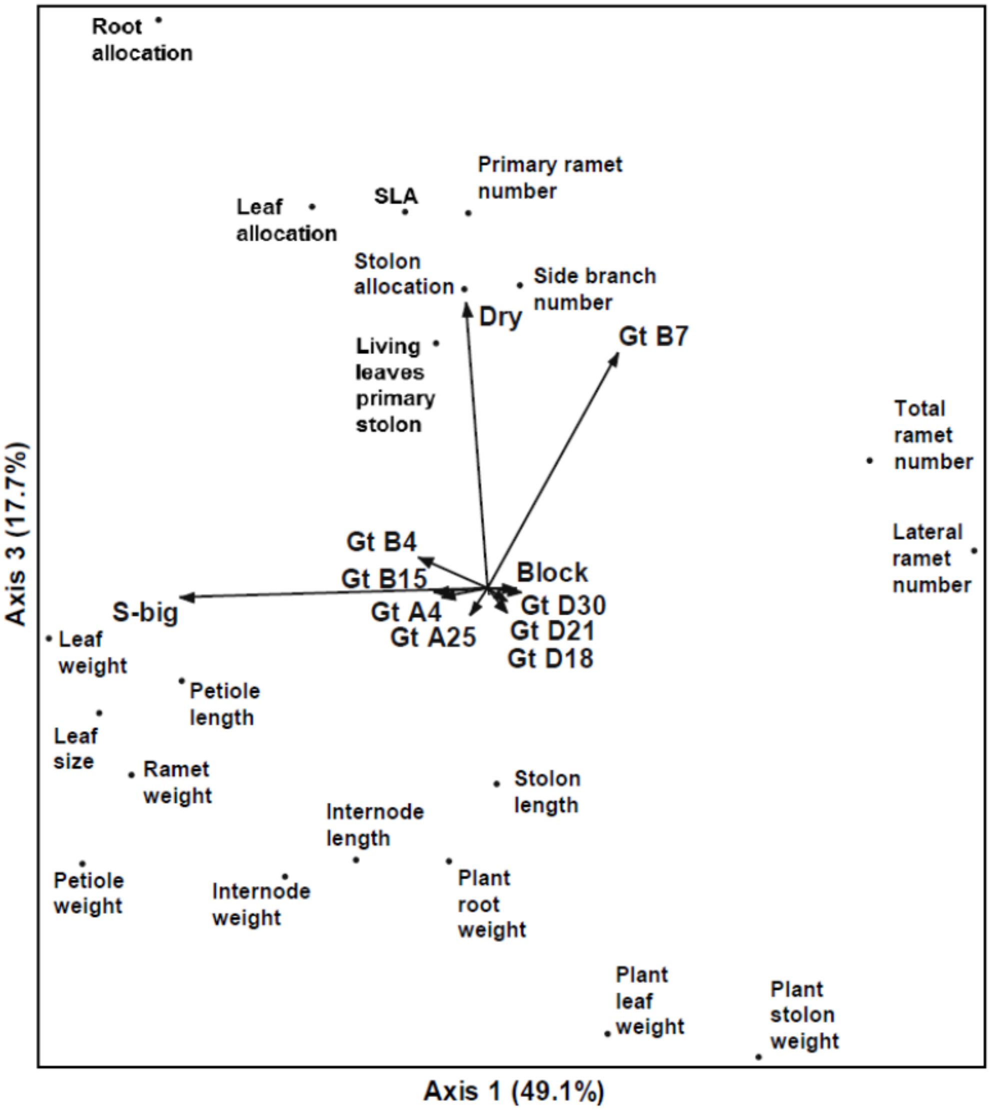

Supplementary file Figure S-6. Ordination diagram of axes 1 and 3 of a Correspondence Analysis of plant traits of eight genotypes of *Trifolium repens* with big (4) or small (4) ramets grown in three different competitive settings (within-genotype, within functional group, and between functional groups), both water treatments. Genotype identities, ramet size class, drought treatment, competitive setting (not visible in graph) and block added as passive variables.

2.2 Supplementary Tables

Supplementary File Table S-1. Correspondence Analysis of plant traits of eight genotypes of *Trifolium repens* with big (4) or small (4) ramets) grown in three different competitive settings (within-genotype, within functional group, and between functional groups) well-watered treatment. Pearson and Kendall correlations with ordination axes 1-3 of genotype identities, ramet size class, drought treatment, competitive setting (not visible in graph) and block.

	axis 1			axis 2			axis 3		
	r	r-sq	tau	r	r-sq	tau	r	r-sq	tau
S-big	-0.57	0.325	-0.491	-0.135	0.018	-0.17	0.484	0.235	0.419
Gt A25	-0.147	0.022	-0.113	0.136	0.018	0.11	0.17	0.029	0.165
Gt A4	-0.217	0.047	-0.213	-0.061	0.004	-0.075	0.289	0.084	0.256
Gt B15	-0.244	0.059	-0.207	-0.035	0.001	-0.06	0.23	0.053	0.206
Gt B4	-0.258	0.066	-0.211	-0.238	0.057	-0.227	0.046	0.002	0.011
Gt B7	0.417	0.174	0.307	-0.436	0.19	-0.355	-0.476	0.226	-0.311
Gt D18	0.149	0.022	0.135	0.214	0.046	0.196	0.021	0	-0.004
Gt D21	0.122	0.015	0.139	0.299	0.089	0.284	-0.172	0.03	-0.197
Gt D30	0.169	0.029	0.157	0.123	0.015	0.127	-0.1	0.01	-0.118
comp F	-0.033	0.001	-0.019	-0.06	0.004	-0.062	-0.019	0	-0.009
comp G	-0.058	0.003	-0.033	0.003	0	-0.008	-0.009	0	-0.003
comp H	0.078	0.006	0.045	0.049	0.002	0.06	0.024	0.001	0.011
Block	0.189	0.036	0.16	0.194	0.038	0.14	0.013	0	0.016

Supplementary file Table S-2. Correspondence Analysis of plant traits of eight genotypes of *Trifolium repens* with big (4) or small (4) ramets grown in three different competitive settings (within-genotype, within functional group, and between functional groups), drought treatment. Pearson and Kendall correlations with ordination axes 1-3 of genotype identities, ramet size class, drought treatment, competitive setting (not visible in graph) and block.

	axis 1			axis 2			axis 3		
	r	r-sq	tau	r	r-sq	tau	r	r-sq	tau
S-big	0.658	0.433	0.563	0.123	0.015	0.181	-0.257	0.066	-0.208
Gt A25	0.145	0.021	0.11	-0.089	0.008	-0.063	-0.242	0.058	-0.214
Gt A4	0.274	0.075	0.239	0.084	0.007	0.13	-0.25	0.063	-0.206
Gt B15	0.249	0.062	0.223	-0.106	0.011	-0.071	-0.061	0.004	-0.043
Gt B4	0.333	0.111	0.284	0.296	0.088	0.278	0.158	0.025	0.143
Gt B7	-0.328	0.108	-0.249	0.561	0.314	0.395	0.305	0.093	0.202
Gt D18	-0.182	0.033	-0.17	-0.256	0.066	-0.229	-0.041	0.002	-0.034
Gt D21	-0.222	0.049	-0.195	-0.31	0.096	-0.277	0.118	0.014	0.125
Gt D30	-0.254	0.065	-0.228	-0.171	0.029	-0.154	0.006	0	0.02
comp F	0.009	0	-0.002	0.007	0	0.025	0.039	0.002	0.041
comp G	-0.021	0	-0.021	-0.017	0	-0.008	-0.002	0	-0.015
comp H	0.01	0	0.02	0.009	0	-0.015	-0.032	0.001	-0.022
Block	-0.206	0.043	-0.169	-0.096	0.009	-0.055	0.306	0.093	0.217

Supplementary file Table S-3. Correspondence Analysis of plant traits of eight genotypes of *Trifolium repens* with big (4) or small (4) ramets grown in three different competitive settings (within-genotype, within functional group, and between functional groups), complete dataset. Pearson and Kendall correlations with ordination axes 1-3 of genotype identities, ramet size class, drought treatment, competitive setting (not visible in graph) and block.

	axis 1			axis 2			axis 3		
	r	r-sq	tau	r	r-sq	tau	r	r-sq	tau
S-big	-0.583	0.34	-0.504	0.315	0.099	0.267	-0.104	0.011	-0.074
Gt A25	-0.138	0.019	-0.106	0.005	0	0.001	-0.174	0.03	-0.148
Gt A4	-0.226	0.051	-0.21	0.198	0.039	0.173	-0.106	0.011	-0.079
Gt B15	-0.245	0.06	-0.216	0.04	0.002	0.026	-0.068	0.005	-0.035
Gt B4	-0.276	0.076	-0.233	0.233	0.054	0.205	0.185	0.034	0.145
Gt B7	0.381	0.145	0.277	0.191	0.037	0.161	0.511	0.261	0.378
Gt D18	0.151	0.023	0.143	-0.186	0.034	-0.158	-0.165	0.027	-0.133
Gt D21	0.148	0.022	0.152	-0.309	0.095	-0.248	-0.121	0.015	-0.088
Gt D30	0.195	0.038	0.182	-0.166	0.028	-0.154	-0.065	0.004	-0.044
comp F	-0.024	0.001	-0.011	0.014	0	0.018	0.035	0.001	0.03
comp G	-0.023	0.001	-0.009	-0.014	0	-0.011	-0.01	0	-0.013
comp H	0.041	0.002	0.017	0	0	-0.007	-0.022	0	-0.015
Block	0.183	0.033	0.157	-0.306	0.042	-0.148	0.023	0.001	0.011
Dry	-0.156	0.024	-0.127	-0.512	0.262	-0.424	0.561	0.315	0.487
